# The use of commercial food purchase data for public health nutrition research: A systematic review

**DOI:** 10.1371/journal.pone.0210192

**Published:** 2019-01-07

**Authors:** Lauren Bandy, Vyas Adhikari, Susan Jebb, Mike Rayner

**Affiliations:** 1 Nuffield Department of Population Health, University of Oxford, Oxford, United Kingdom; 2 Nuffield Department of Primary Care Health Sciences, University of Oxford, Oxford, United Kingdom; McMaster University, CANADA

## Abstract

**Background:**

Traditional methods of dietary assessment have their limitations and commercial sources of food sales and purchase data are increasingly suggested as an additional source to measuring diet at the population level. However, the potential uses of food sales data are less well understood. The aim of this review is to establish how sales data on food and soft drink products from third-party companies have been used in public health nutrition research.

**Methods:**

A search of five electronic databases was conducted in February-March 2018 for studies published in peer-reviewed journals that had used food sales or purchase data from a commercial company to analyse trends and patterns in food purchases or in the nutritional composition of foods. Study quality was evaluated using the National Institutes of Health (NIH) Quality Assessment Tool for Cohort and Cross-Sectional Studies.

**Results:**

Of 2919 papers identified in the search, 68 were included. The selected studies used sales or purchase data from four companies: Euromonitor, GfK, Kantar and Nielsen. Sales and purchase data have been used to evaluate interventions, including the impact of the saturated fat tax in Denmark, the soft drink and junk food taxes in Mexico and supplemental nutrition programmes in the USA. They have also been used to identify trends in the nutrient composition of foods over time and patterns in food purchasing, including socio-demographic variations in purchasing.

**Conclusion:**

Food sales and purchase data are a valuable tool for public health nutrition researchers and their use has increased markedly in the last four years, despite the cost of access, the lack of transparency on data-collection methods and restrictions on publication. The availability of product and brand-level sales data means they are particularly useful for assessing how changes by individual food companies can impact on diet and public health.

## Introduction

Diet-related diseases, including cardiovascular disease, cancer and diabetes, are the leading cause of mortality and morbidity globally [1] with poor diet resulting in one in five deaths in 2016 [2]. The prevalence of diet-related disease is rising and this is partly attributable to an increase in the availability and subsequent purchase and consumption of foods high in energy, saturated fat, sugar and salt. In many high-income countries, diets are now dominated by highly processed foods, which represent up to 79% of mean energy intakes [3]. In 2017, the retail value of sales of processed foods and soft drinks was estimated to be US$2.7 trillion worldwide, rising by US$550 billion in the last decade [4]^.^

To achieve the World Health Organization target to halt the rise in obesity and diabetes [5], dramatic changes are needed to food environments–including action by the food industry—to motivate and sustain healthier diets. At the UN General Assembly High Level Meeting on the Prevention and Control of Non-communicable Diseases in 2011, Heads of State and Government were asked to call upon the private sector to consider promoting and producing foods that are more consistent with a healthy diet, including through reformulation [6]. Since then, a number of governments worldwide have been working with the food industry to drive change. For example, in the United Kingdom, the government established the Public Health Responsibility Deal from 2011–2015, a public-private partnership that saw businesses sign up to voluntary public health targets [7] and in 2017/18 Public Health England introduced specific sugar and calorie reduction targets [8][9].

However, measuring the effectiveness of policies to change the food environment is difficult as research methods to monitor the nutritional quality of the food supply or the impact of the changes being made by individual manufacturers are poorly developed. Researchers have typically relied on food balance sheets and agricultural and economic data to monitor the food supply or dietary surveys to monitor consumption, but these have their limitations. They look at the production, import, export and stocks of commodities and their domestic utilisation, but provide no details on specific products or on the sub-populations who consume them [10]. Dietary surveys often have a small sample size, are resource-intensive, subject to misreporting and rarely provide information on the brand of products being consumed [11].

Commercial data provided by third-party companies have long been by economists, business schools and health researchers, including in research relating to tobacco use [12], beauty and personal care products [13] and firearms [14]. Commercial food sales and purchase data are a complimentary source of information for researchers looking at dietary patterns. Although much happens between a food item being bought and it being consumed, including food preparation, distribution among household members and waste, food purchase data are a good indicator of diet. The value and volume sales of food and beverage products are available from private market research companies and predominantly used by the food industry to track product performance, market shares, sales, brand loyalty and the success of advertisement campaigns and promotions [15]. These data are usually presented as annual totals at the national level, or split by product category, company, brand and sociodemographic factors. Food sales data measures how much of a product has been sold from the supplier side, where as food purchase data measures how much of a product has been purchased by consumers. While neither sales nor purchase data measure exactly what individuals have consumed, they have the potential to be used by public health nutrition researchers and policy makers to monitor changes being made to the nutritional quality of the food supply.

The main aim of this review is to systematically establish how sales and purchase data for food and non-alcoholic drink products from third-party companies have been used in public health nutrition research and to consider how this type of data may contribute to the advancement of knowledge to accelerate improvements in dietary intake. This review will provide a synthesis of the principal research questions, the methods employed and the conclusions drawn with the objective of ascertaining how food sales data can best be utilised by researchers in the future to improve the diet of the population.

## Methods

### Search identification

In March 2018, five databases (PubMed, Web of Science, Psych Info via Ovid, Scopus and Business Source Complete) were searched for terms relating to the names of six companies known as providers of relevant data with no restrictions on date: Euromonitor, Kantar, Mintel, GlobalData, Canadean and IRI in the UK and globally. Search terms related to food or beverage sales data, consumption, household panel, retail, supermarket, nutrition and diet were also included to ensure that studies that use data from a company that was not listed or studies that do not name a specific company in the abstract were included. For the full search terms, see the published PROSPERO protocol (CRD42018091421)

### Study screening and eligibility

Only studies that use food and non-alcoholic drink sales data in a way relevant to public health nutrition were included. All populations in all settings, including household, national, multi-national or global level, were included and all interventions and exposures were deemed of interest.

The primary study outcomes for eligibility were:

1. Value or volume sales of food or beverage products e.g. sales of fruit and vegetables or sales of confectionery
2. Volume sales of nutrients e.g. calories, grams of sugar, fat, salt per day

Only papers that used data from a commercial provider were included–papers that use data directly from individual grocery stores, supermarkets and other retail outlets were excluded. This is because this review focussed on commercial data that is readily available to researchers in public health nutrition. Accessing sales data from stores directly can involve significant negotiations and contracts. Only articles published in peer-reviewed journals were included. There was no restriction on date and only papers written in English were included.

Identified records were exported to Mendeley (Desktop version 1.17.13) and duplicates were removed. The records were then exported to CSV and saved as an MS Excel worksheet for the management of the screening. One investigator (LB) first screened by title, and then two investigators (LB and VA) independently reviewed the included titles, abstracts and methods.

### Data extraction

Data extraction was conducted for all included studies and completed by two investigators (LB and VA), with a 10% check done by a third investigator (MR). Any discrepancies between the investigators were discussed and LB made the final decision. The following data were extracted for each article where relevant: study ID (first author and date), title, research aims/questions, setting (geographical location), population demographics, study design, exposure and outcome variables measured, source of sales data, source of nutrient composition data, classification system used for definitions of “healthy” or “unhealthy” foods, summary of key findings.

### Quality appraisal

Two reviewers (LB and MR) independently assessed risk of bias and study quality using the National Institutes of Health (NIH) Quality Assessment Tool for Cohort and Cross-Sectional Studies. Any discrepancies between the two investigators were discussed and a third opinion (VA) sought if an agreement could not be made. LB made the final decision.

## Results

**Fig 1:**
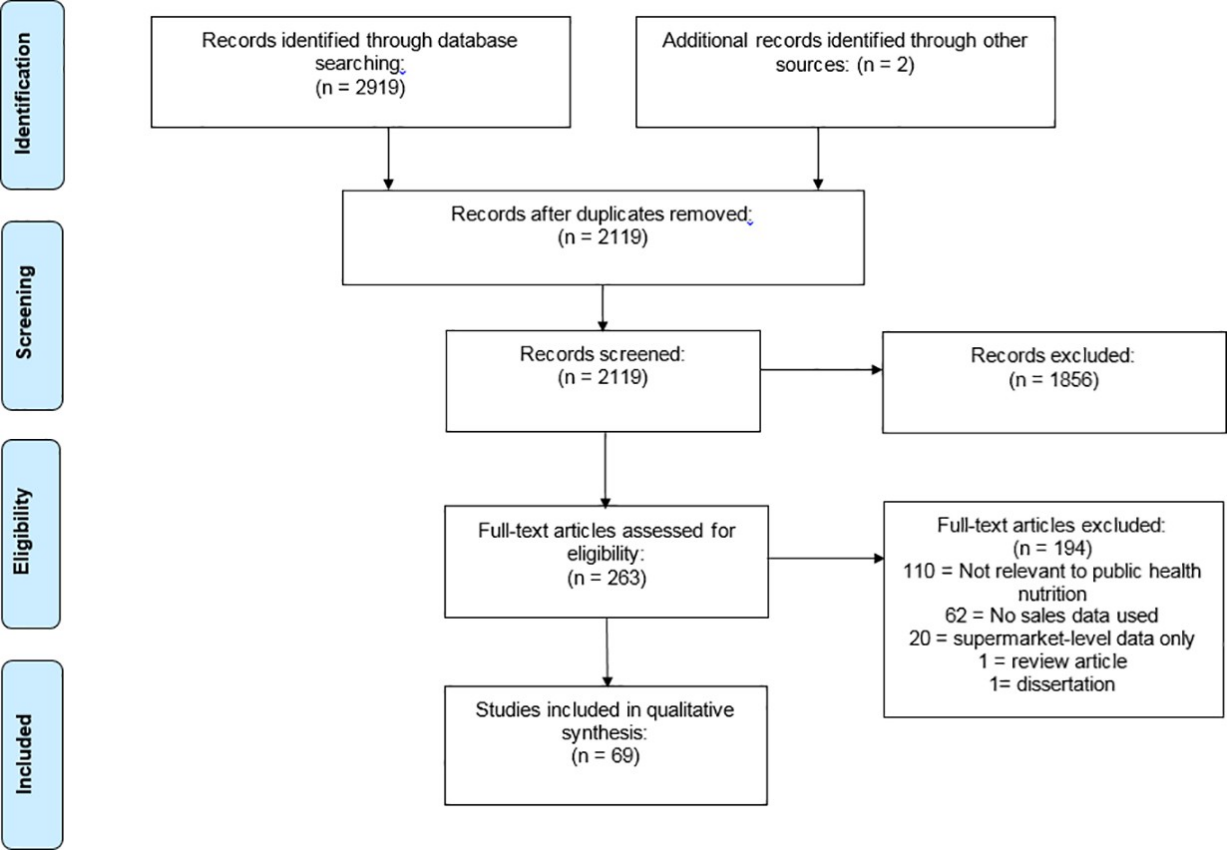
Study selection flow chart.

A total of 2920 potential articles were identified during the search, from which 69 were selected for inclusion. Fig 1 shows a flowchart of the literature search and study selection.

### Data sources

**Fig 2:**
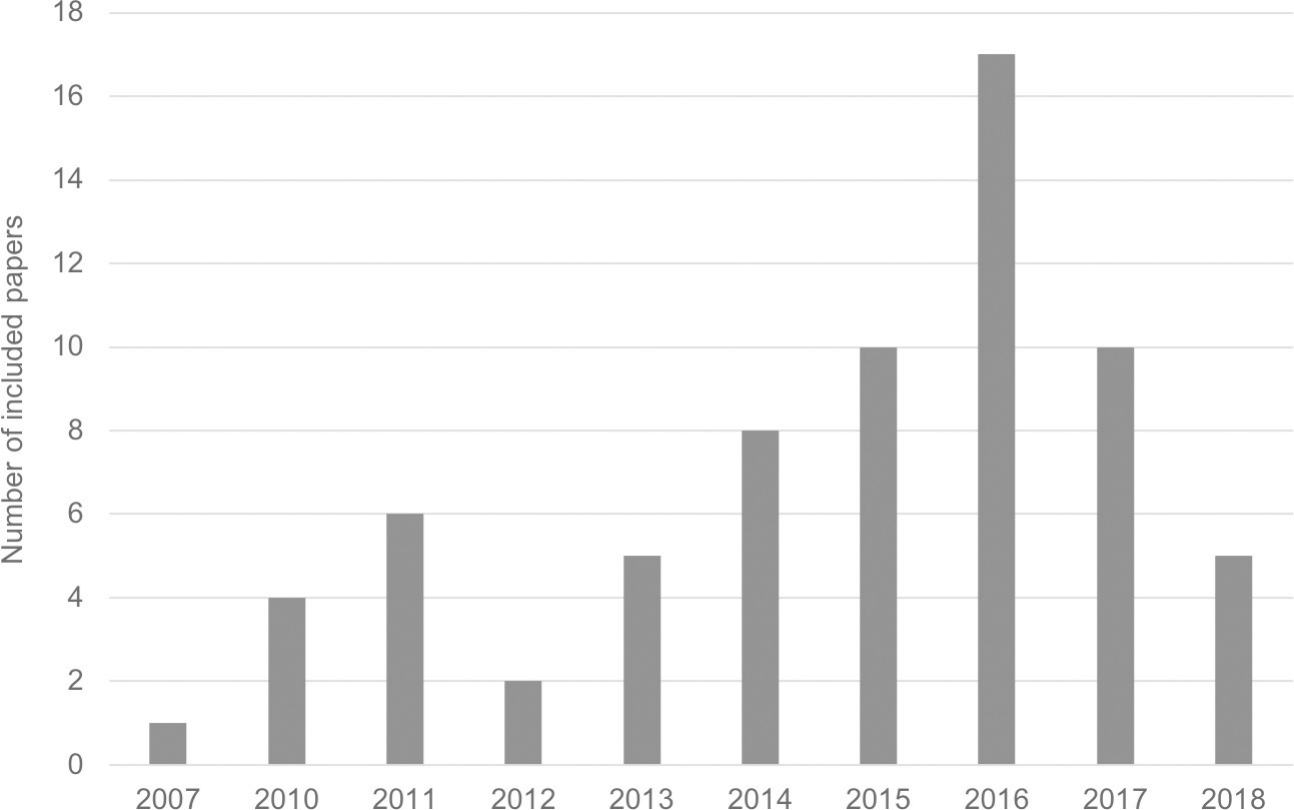
The number of studies that used food sales data by year of publication.

Four commercial sources of sales and purchase data were identified. These were: Euromonitor, GfK, Kantar and Nielsen. Euromonitor provided data on what has been sold by food and beverage companies and GfK, Kantar and Nielsen provided data on what has been purchased by household panels. The majority of papers (n = 56) used data from either Nielsen or Kantar. A clear geographical pattern was identified, with all of the studies in the USA using Nielsen data, all the studies in the United Kingdom and France using Kantar data, all of the Danish studies using GfK data and those that looked at multiple countries or had a global outlook used Euromonitor data. The first study that used food sales data was published in 2007. From 2011 until 2016, the number of studies increased year-on-year. A full breakdown of the sources of data and their geographies used can be found in Table 1 and the number of studies published by year is represented in Fig 2.

**Table 1:** Number of papers published that use food sales data by source company and country of research.

	Data source company				
Country	Nielsen	Kantar	GfK	Euromonitor	Total
USA	29	0	0	0	29
UK	0	13	0	0	13
France	0	6	0	0	6
Denmark	0	0	5	0	5
Multiple countries/global	0	0	1	4	5
Mexico	3	0	0	0	3
Australia	2	0	0	0	2
New Zealand	2	0	0	0	2
Canada	1	0	0	0	1
India	0	0	0	1	1
Netherlands	0	0	1	0	1
Sweden	0	0	1	0	1
**Total**	**37**	**19**	**8**	**5**	**69**

Data from all four companies were used to analyse the volume and value sales of food or beverage products, with GfK, Kantar and Nielsen also providing data on price. Forty-seven of the papers used nutrient composition data alongside sales/purchase data in their analysis, with data from GfK, Kantar and Nielsen being used to translate product sales into the purchases of nutrients. Kantar and GfK provided paired sales and nutrition composition data for 11 of the included studies. Composition data from other sources, including national composition databanks and other commercial companies, were used in the remaining 36 papers that combined sales/purchase and composition data. A summary of the different types of data used by each data source is given below in Table 2.

**Table 2:** Summary of data types used in the included studies by company.

Company	Research method	Volume sales	Value sales	Nutrient purchases	Product price data	Granularity of data	Provided composition data
Euromonitor	Secondary data collection	Yes	Yes	No	No	Brand level sales only	No
GfK	Household panel	Yes	Yes	Yes	Yes	Individual product sales	Yes
Kantar	Household panel	Yes	Yes	Yes	Yes	Individual product sales	Yes
Nielsen	Household panel	Yes	Yes	Yes	Yes	Individual product sales	No

Note: this table summarises the data types used in the included studies only. The data providers should be contacted directly for information on coverage and other data types.

Methods of data collection for each data provider differed, although GfK, Kantar and Nielsen all use household panels to collect their data used in the included studies. Participating households are given a handheld scanner and asked to scan the barcode of every individual grocery product that they purchase, collecting the product name, pack size, price and location of the retailer where the items were purchased. Incentives for participation, in the form of high-street vouchers, are used by all three companies. Euromonitor do not undertake primary data collection but instead undertake ‘desk research’–that is they use economic indicators, publicly available information, trade news, company reports and data shared in trade interviews as the basis for their data set [4].

### Quality of studies

The study quality assessment tool for observational cohort and cross-sectional studies was applied to the 69 papers. Overall, the studies were found to be of a similar quality, but all had limitations.

The 64 studies that used purchase data derived from household panels were limited by their recruitment process. The recruitment of the households to the panels was undertaken by each commercial company, meaning the authors had no control over this process, the incentives for participation used, and could not report on recruitment bias and drop-out rates. Few of the studies mentioned these limitations in their discussions and few raised questions on other limitations of the data, including the fact that out of home purchases were not covered. This may have been due to publication restrictions imposed by the data provider and therefore cannot be overcome by the authors of the individual studies themselves.

Scanned data, while more objective than other methods such as food diaries and 24-hour recalls, are still not 100% accurate for two main reasons. Firstly, there is a reliance on participants scanning all products, including ones they have purchased when they do not have immediate access to the scanner. Secondly, products without barcodes–such as those purchased at a market or in a restaurant—cannot be scanned and therefore the data is liable to be under-reported. Euromonitor sales data is not derived from primary data collection but based on desk research (i.e. secondary data collected from the trade press and trade interviews); the exact sources of the data are not made available and so transparency, reliability and accuracy are all open to question. None of the five studies included in this review that use Euromonitor sales data discussed these limitations.

### Objectives and key findings of the included studies

For the purpose of this analysis, the studies have been split into three groups based on their study design: longitudinal studies (n = 37), cross-sectional studies (n = 22) and modelling of future scenarios (n = 10). These are further subdivided based on whether they are purely observational or are studies reporting the outcome of an intervention study. Further details on each study, including objectives, data sources, measured variables and a summary of key findings, can be found in S1 Table.

### Longitudinal intervention studies

14 papers were of longitudinal design and examined interventions. Two of these papers looked at marketing interventions. One examined the impact of breakfast cereal coupons on the nutritional quality of purchases, finding that breakfast cereals purchased with a manufacturer or retail coupon had a higher sodium and sugar content [16]. The second analysed the impact of Coca-Cola’s sponsorship of the Winter Olympic Games in 2006 and Summer Olympic Games in 2008, with results suggesting consumers chose Coca-Cola more frequently than Pepsi during this three-year time period [17].

Twelve papers examined public health interventions. Four papers evaluated national-level taxes; two in Mexico using Nielsen data and two in Denmark using data from GfK. One paper used Nielsen data on the purchases of soft drinks to evaluate the excise tax on sugar-sweetened beverages in Mexico that was introduced in 2013 [18]. The study found that over 2012 to 2014, purchases of taxed beverages had declined by an average of 6%, with higher reductions seen among households classified as low socioeconomic status [18]. Another paper used a similar data set from Nielsen and similar methods to evaluate the tax on non-essential energy dense foods [19]. It found a 5% decline in the mean volume of purchases of taxed foods over 2012 to 2014, with a 10% decline in low socioeconomic households [19]. Two papers used data from GfK to analyse the impact of the 2011–2012 Danish tax on saturated fat. One analysed purchases of butter, blends, margarines and oils from 2008 to 2012 and found that the tax led to a reduced consumption of these products by 10–15%, with a shift in purchasing from discount retailer chains [20]. The second paper used GfK data on processed foods in combination with nutrient composition data from the Danish Food Composition Databank to estimate the effects of the tax on packaged food consumption and model these effects on mortality from NCDs [21]. It found the tax led to a 4% reduction in the consumption of saturated fat, an increased vegetable consumption and increased salt consumption, with a modelled reduction in mortality showing that 123 lives saved annually [21].

Three papers in the United States used purchase data to analyse the effect nutritional assistance programmes had on the composition of household diets. Two papers looked at the Special Supplemental Nutrition Assistance Program for Women, Infants and Children (WIC) and profiled the purchases of households before and after major policy revisions in 2009. The results showed that WIC participation was associated with increased purchases of wholegrains compared to non-participating households [22] and that the revisions had led to a significant reduction in purchases of calories, sodium, saturated fat, sugar, refined grains, high-fat milk and sugar-sweetened beverages (SSBs), and an increase in fruit and vegetables [23]. A third paper looked at the Supplemental Nutrition Assistance Program (SNAP) and found that SNAP households had reduced mean purchases of fruit and vegetables and fibre and an increase in junk food products, saturated fat and salt [24] compared to non-participating households.

One paper looked at what impact banning the sale of carbonated beverages in schools had in households with school-age children in the US [25]. Results showed that when high schools banned the sales of sodas, the affected children increased their household consumption of non-diet sodas by 3.4 cans per month, offsetting the impact of the reduction in consumption in schools [25].

Four studies evaluated voluntary initiatives. Two looked at the impact of front-of-pack (FOP) labelling. One study using GfK data showed that consumers paid higher prices for products that carried the Choices and Keyhole labels in the Netherlands and Denmark respectively [26] and another looked at the impact of the Health Star Rating FOP label in New Zealand using Nielsen data [27]. It found that products displaying the label had a higher energy density but lower saturated fat and sugar and sodium content [27]. One paper looked at how the introduction of voluntary sodium reduction targets in the UK impacted on sodium consumption and the sodium content of food products between 2006–2011 [28]. Using Kantar purchase and composition data, it found sodium consumption fell by 7% overall, with snack foods, dairy products and sauces and spreads seeing the greatest reductions [28]. Another paper evaluated the effect of the calorie reduction pledge of the Healthy Weight Commitment Foundation in the US [29]. The results using Nielsen data showed that participating companies’ products represented 25% of calories consumed in the US in 2007. They estimated that the calorie reduction pledge target of 1.5 trillion calories was equivalent to 0.8% of total calories purchased from packaged food and beverages [29].

### Longitudinal observation studies that focussed on nutrient composition

Five papers looked at trends in nutrient composition of foods over time. Two of these used Kantar data to track the nutritional content of foods in France, with one finding that the sales-weighted sodium content of potato chips and breakfast cereals had fallen by 6.7–11.1% and 7.3–9.7% respectively from 2008 to 2011 [30] and another finding that the energy density of foods overall had increased, but that total caloric purchases had fallen by 6.7% over 1969–2010 despite the energy density of foods increasing, overall purchase of energy had fallen by 6.7% [31]. Two studies conducted in the US analysed the nutritional quality of food purchases based on store-type. One found there had been significant reduction in energy, sugar and salt densities of packaged food products from Walmart from 2000–2013 [32], and the other found that packaged food products sold in warehouse-club, convenience and mass-merchandise stores had poorer nutrient densities than other store types [33]. Another paper compared the nutritional quality of foods with their degree of processing and convenience, with results suggesting that highly-processed foods have a higher sugar, saturated fat and sodium content compared to less-processed foods [34].

Three papers looked at trends in individual components of foods as opposed to the overall nutritional quality. One tracked the use of sweeteners in packaged food and beverages, with results showing that 73% of products in the US contained an added caloric sweetener [35]. Another analysed the sodium content of packaged foods, finding that the amount of sodium purchased from packaged foods fell by 396mg/day per capita from 2000 to 2012 and a third paper analysed the nutritional content of an individual food product, grain-based desserts, finding a reduced saturated fat and sugar contents of these products led to a reduction in their energy density (from 433kcal/100g in 2005 to 422kcal/100g in 2012).

### Longitudinal observational studies that focussed on food purchasing

Fifteen observational studies focused on the purchasing patterns of foods.

Six papers examined the relationship between sociodemographic factors and food purchasing patterns and behaviours. One paper examined the association between ethnicity, income and store preference in the US, with results showing that there was no association between these factors, except for low income non-Hispanic black households, which were less likely to use mass-merchandise stores [36]. Two looked at how food purchase patterns vary with race and ethnicity in the US, with one study finding that black households purchased less processed and ready to eat foods and more basic foods including oils and sugar when compared to white households [37], and another study found that total energy purchased declined from 2003–2013, with smaller reductions seen in black and low-income households compared to white, high income households [38]. Another US paper found that purchases of energy in households with pre-school aged children declined over 2000–2011, but smaller reductions were seen those households with a low income [39]. One study that took place in Mexico found that low socioeconomic status households purchased more food and beverage products defined as unhealthy but also saw the largest reductions in purchases of these products from 2012–2014 [40]. Another study in Denmark looked how unemployment affected food purchase behaviours, with findings showing that medium-term unemployment led to a reduction in food expenditure and a reduction in the amount of animal products, fats and proteins purchased [41].

Three papers focused on the purchasing patterns of soft drinks. One analysed Euromonitor data in 44 low and middle income countries and found that tariffs on sugar-sweetened beverages were inversely, but not significantly, associated with consumption [42]. The second paper used Nielsen data to look at the trends in soft drink sales in Australia over 15 years, concluding that volume sales had fallen from 8.4kg per capita a year to 6.2kg per capita per year [43]. A third paper looked at the impact that soft drink purchases had on the nutrients purchased from other foods, with results showing that for each 1 serving/day increase in consumption of sweetened beverages purchases of total calories, carbohydrates, total sugars and total fats also increased [44].

Three papers used purchase data as a basis for modelling consumer preferences. One used Nielsen data in Canada to model consumer preferences for meat, finding that health preferences triumphed over price [45] and another using GfK data showed that Danish consumers who preferred high-fat milk did not change their preferences after reading health information on fat intake [46]. A third modelled the short- and long-term effects of the first choice of a low-fat product on the subsequent purchases of calories and product volume. Results showed that purchasing low-fat crisps/chips leads to an increase in overall caloric purchases in the short- and long-term [47].

The remaining three papers looked at the impact of the 2007–2010 global recession on dietary habits. One looked at how changes in the relative price of foods affected nutrient purchases in the UK in 2007–2008 and found that price changes led to a decline in the nutritional quality of household food baskets [48]. A second paper in the UK looked at how adjusting for waste affected the trend in declining energy purchases from foods during the recession [49]. Energy purchases were found to decrease from 8.6MJ/adult to 8.2MJ/adult equivalent per day between 2007 and 2012, although this decrease was not significant after adjusting for waste [49]. A third paper conducted in the US using Nielsen data also found that energy purchases had declined from 2003–2010 but increased slightly during the recession, with a 1% increase in unemployment associated with a 1.6–4.1 kcal per capita per day increase in the total energy purchased [50].

### Cross-sectional observation studies that focussed on nutrient composition

One paper used food purchase data from Kantar to assess the nutrient content of bakery and breakfast products. They predicted that reformulating the levels of sugar, fat, fibre and salt in products of the lowest nutritional quality could lead to a significant variation in an individual’s nutrient intake [51]. Another paper used Kantar data in the UK to focus on the sodium content of products purchased, with table salt, processed meat and bakery products being the top contributing categories to sodium intake [52]. One paper used US Nielsen data to establish if there was a difference in the nutritional quality of foods purchased based on the stores that households shop in [33], with results showing that there was no difference between the nutrient content of food products purchased primarily in grocery stores compared to other store types, including convenience and mass-merchandise stores [33].

Two other papers focused on the development or comparison of methods, rather than the results of the purchase data themselves. One paper compared the results of an in-store survey with that of food purchase data, finding that the mean sodium and sugar content of the products recorded in the in-store survey were higher than those recorded in the food sales data [53]. Another outlined how food purchase data can be paired with nutrition composition data taken from multiple sources in the US, allowing for a monitoring tool to be developed that tracks the nutritional quality of foods “from factory to fork” [54].

### Cross-sectional observation studies that focussed on food purchasing

Seven papers describing cross-sectional studies examined associations between the sociodemographic factors of household food and beverage purchases. Three examined the adherence of households to dietary guidelines. One paper looked at the affordability of meeting the MyPyramid fruit and vegetable scheme. They found that in 2008, a wide variety of fruit and vegetables were available at $0.40–0.50 per cup equivalent, meaning that low-income households would have to have spent 40–50% of their food budget on fruit and vegetables to meet the target amount [55]. Two studies conducted with Kantar data in Scotland found that households with a higher level of deprivation were less likely to achieve the revised Scottish Dietary Goals [56][57]. Another paper that used Kantar data in Scotland analysed the expenditure on fresh foods and fruits and vegetables across urban and rural households [58]. They found that rural households spent the most on fresh foods, fruits and vegetables but prices in urban locations were significantly higher, suggesting that factors other than availability and price were causing the difference [58]. Two UK studies using the same Kantar dataset from 2010 also looked at food purchase patterns in households in relation to sociodemographic factors. One looked at store preferences and found that although supermarket choice and shopping behaviour were both associated with the healthfulness of purchases, neither appeared to be associated with socioeconomic differences [59]. A second paper aimed to explore how food expenditure mediates socioeconomic inequalities in the healthiness of household food choices and found that higher social class was associated with higher expenditure on foods that were healthy, suggesting that lower spending on food leads to less healthy choices for lower socioeconomic groups [60].

Three studies with a cross-sectional design used Euromonitor data to analyse food sales by company. One study looked at the value sales and market shares of member companies of the International Food and Beverage Alliance (IFBA). It concluded that while the global top 10 soft drink companies account for half of global sales of soft drinks, the top 10 packaged food companies represent only 15% of global food sales [61]. This means that public health commitments from these companies is likely to have a much smaller public health impact [61]. Another study identified the main companies contributing to food and beverage sales in 12 countries in Asia, finding that sales of ultra-processed foods from a limited number of companies was driving the nutrition transition in the region [62]. The third study used sales data paired with nutrition information collected from the packaging and nutrient profiling to assess the healthiness of India’s largest food companies’ product portfolios [63]. Overall, they found that the healthiness of products was low, with significant variation within the same product category [63].

One paper used sales data from Euromonitor and looked at the sale of soft drinks and prevalence of obesity and type 2 diabetes in 75 countries, finding a positive association between the two [64]. Another study used sales data to examine the patterns of food and nutrient purchases by BMI, finding that individuals with overweight and obesity consume more energy at all ages and 20% more fat than those individuals with a normal weight [65].

One US study looked at how the food purchases of diet soda drinkers compared to those of regular soda drinkers. The results showed that diet soda consumers spent a smaller proportion of their total annual grocery spend on foods higher in energy, such as fruit juice and dairy products, and spent a higher proportion on lower fat versions of foods, such as dairy products, frozen entrees and salad dressings [66]. One study examined patterns in breakfast cereal purchases in Denmark. They showed that breakfast cereal purchases were very habitual, although the persistence of the behaviour was weaker in households with one or more children, compared to single-adult households [67]. Another study focussed on how breakfast cereal purchasing patterns varies depending on sociodemographic makeup of households in the US, with results showing that low income African-American and Asian households and those with one or more children purchased the most ready-to-eat breakfast cereals [68]. An analysis of household sociodemographic trends in relation to price promotions in the UK found that sales of promotional items was higher in low SES households compared to high SES households. However, there was no significant SES gap in the purchase of less-healthy foods on promotion [69]. Another paper used food sales data to examine consumer demand patterns for products with claimed nutritional benefits. It found that consumers are more sensitive to price decreases and less sensitive to price increases for both healthy and unhealthy food and that these sensitivities are greater for products with no nutritional benefit, supporting the hypothesis that products with nutritional benefits have a higher brand equity than those without [70].

### Scenario modelling studies

Thirteen papers used purchase data to model different scenarios, with seven papers looking specifically at soft drink tax scenarios in Australia [71], the UK [72][73] or USA [74][75][76][77]. All reported that an increase in the price of selected soft drink products led to a reduction in their volume consumption. Three papers used Nielsen data to model the impact of a 20% tax on selected soft drinks on body weight. The results showed a 20% tax led to weight loss ranging from 0.32kg/capita/year [75], 0.4kg/capita/year [71] to 0.7–1.16kg/capita/year (71). Three examined the impact of a tax by household income, with two finding the greatest reductions in beverage consumption and body weight for low income households [71][72] and the other for middle income households [75]. Two papers looked specifically at the impact of soft drink taxes on the diets of preschool children using UK Kantar and US Nielsen data respectively, with both finding a tax was associated with reductions in energy purchased from beverages but not with an improved total dietary intake [76][77]. One paper used Kantar data in the UK and concluded a tax on soft drinks would have a minimal impact on calorie consumption (70).

Three other papers modelled the introduction of taxes on food products. One used US Nielsen data and found that the introduction of a sodium tax on meats eaten at lunchtime reduced the consumption in terms of both products and sodium [78]. The second, which used Kantar data to consider a tax on French packaged food products based on their fat and sugar content, reported small and ambiguous effects [79]. The third paper also used Kantar data. It compared the effects of a tax on standard yoghurts, fromage blanc and dessert yoghurts to front-of-pack nutritional labelling in France. It found that both policies were equally effective at reducing purchases of fat from these products at around 7% and 8% respectively, but the tax had an economic benefit to the state that the labelling policy did not [80].

## Discussion

This review has drawn together the wide range of studies using food purchase and sales data for public health nutrition research, identifying a range of applications including the monitoring of the nutrient content of foods over time, analysing dietary patterns by demographic factors, evaluating existing food and beverage taxes and scoring the healthiness of individual companies’ product portfolios. The use of food sales data in academic research has increased rapidly in recent years, with nearly three quarters of the papers included in this review being published in the last four years.

### Strengths and limitations

To the best of our knowledge, this systematic review is the first that has looked at how food sales and purchase data has been used for public health nutrition research. We undertook a comprehensive search including searching business, economics and marketing journals, as well as traditional biomedical and social science journals. The searches, screening and data extraction were done in a systematic and documented manner based on the PRISMA best-practice statement for conducting systematic reviews. However, since this data is mainly used by the food industry to analyse sales performance there are likely to be other studies not included here. In particular, we focused on the published literature in established scientific databases and journals and the grey literature was not explored. Industry reports and policy documents at the national and international level that have used food sales data are not included. Studies that used data directly from retail outlets were excluded. This is because this review focussed on commercial data that is readily available to academics interested in this area of research. Accessing sales data from stores directly can involve significant negotiations and contracts. However, by excluding these papers, this systematic review may have missed papers with similar analyses to those reviewed here.

To assess the quality of studies, we used the National Health Institute’s quality assessment tool for observational cohort and cross-sectional studies. However, some of the elements in this tool were not applicable to the studies that were included, given that few had a traditional exposure/outcome study design. The authors recognise that there may be weaknesses in the quality assessment as a result, but that there were no other established tools that seemed likely to provide a more detailed critical appraisal [81][82].

One common issue that limits the quality of the studies included in this review is that household recruitment to panels is conducted by the data providers. Data companies can and do share information on recruitment, dropout rates and participation incentives, but academic researchers themselves have no control over the recruitment process. Datasets are often incomplete, and gaps are filled by modelled data, again done by the commercial company rather than the researcher, adding to a lack of transparency at how exactly the final datasets were derived. However, these limitations are based on the data sources and cannot necessarily be overcome by the authors of individual studies. A major limitation of food purchase and sales data is that they are often very expensive, limiting their accessibility to academic researchers, unlike national food survey results, which are usually publicly available. Commercial data providers also limit what can be published, including restrictions on detailed methods and company and brand names. These weaknesses in transparency, combined with the high price tag, arguably means that sometimes the negatives of food sales data outweigh the benefits.

There are however some instances in which there are clear benefits to using food purchase/sales data compared to food consumption studies. Most studies included in this systematic review used data collected from hundreds of thousands of households, whereas dietary surveys usually have a smaller number of participants [83]. Purchase and sales data are usually more granular than dietary survey data and include information on price, store location, product size, brand and manufacturer and allow a detailed demographic breakdown. Data from GfK, Kantar and Nielsen are collected and updated continuously, unlike dietary surveys results, where publication is often delayed by a number of months and at a national level, generally not reported more than once a year and often less frequently.

For studies that used GfK, Kantar and Nielsen data, the size of the household panels used varies significantly from study to study; for example, a number of US studies [84][32][85][33] used Nielsen data collected from hundreds of thousands of households, compared to other studies in Europe that analysed the purchases of a just a few hundred households [47][86]. Larger household panels are more likely to generate datasets that more representative of the general population’s dietary habits. Euromonitor does not conduct primary data collection and its data is based on industry research and trade interviews conducted by research analysts.

### Implications of this research

The findings of this review show that food sales data can be used by public health nutrition researchers to measure dietary patterns and monitor the nutrition composition of food products over time. These data can be used as a tool for international comparisons since the commercial companies that provide food sales data are multinational and use the same methods for data collection across countries. This is particularly useful for countries that do not have national dietary surveys, whose results on dietary surveys are poor or whose dietary surveys are carried out irregularly.

Food sales and purchase data are available at a company and brand level, meaning they can be used to monitor the individual contributions companies are making to the food supply. Five studies in this review were identified as using company and/or brand level data. Monitoring how companies react to voluntary policies and greater accountability surrounding the nutritional quality of companies’ product portfolios is an important element of public health nutrition research that is currently underexplored. As well as the academic studies included in this review, there are other initiatives that have already done this, including the Access to Nutrition Index (ATNI). This uses Euromonitor sales data and nutrition information to rank the quality of the world’s top 22 food and beverage companies by the nutritional quality of their products [87]. INFORMAS (International Network for Food and Obesity/NCD Research, Monitoring and Action Support) uses Euromonitor sales data to identify prominent food companies in different sectors as part of their Business Impact Assessment on Obesity and Population-level Nutrition (BIA-Obesity) tool, whereby company policies and commitments to obesity and population health are assessed [88]. Public Health England uses food sales data from Kantar to evaluate progress being made towards the 20% sugar reduction targets they set for selected food categories, although this is being done by food category and not company [89].

Recommendations for future research include reviewing the grey literature, where food purchase and sales data has been used by a host of different actors, including government departments, food manufacturers, marketing companies and investment banks. A review of studies that use food purchase data directly from supermarket stores and chains is also recommended. Further discussions with data providers, researchers who have used these data and economists are also recommended to draw out detailed case-studies on how food sales data can best be used to improve public health.

## Conclusion

The results of this systematic review have shown that food purchase and sales data can be used by public health nutrition researchers to measure dietary patterns, estimate nutritional intake, track the nutritional composition of food products over time, model disease outcomes and evaluate policies. Its use in academic research has increased significantly the last few years and despite its high cost, lack of transparency over household recruitment and imputed data and some restrictions in publication. The use of food sales data has great potential when it comes to evaluating some food policy interventions, including tracking the nutritional quality of the food supply and assessing how individual food companies are responding to mandatory or voluntary targets. However, researchers using these data should be fully aware of their limitations and contextualise their findings accordingly. The authors also call for food sales data providers to be more transparent with their data collection methods to ensure that public health nutrition researchers using their data meet the standards required for peer-reviewed literature.

## Supporting information

S1 Table Summary table of included papers. Includes title, authors, objectives, data types used, variables measured and key findings.
(DOCX)

S1 File PRISMA checklist. (DOC)
