# Supplementary material for: The use of commercial food purchase data for public health nutrition research: A systematic review
Source: PLoS One. 2019 Jan 7;14(1):e0210192. doi: 10.1371/journal.pone.0210192 (PMC6322827; doi:10.1371/journal.pone.0210192)
Supplement: S1 Table — Includes title, authors, objectives, data types used, variables measured and key findings. (DOCX) [file pone.0210192.s001.docx]

| **Author-date** | **Title** | **Objective** | **Geography** | **Demographic** | **Study design** | **Intervention or non-intervention** | **Measuring patterns in food purchases or nutrient content of foods** | **Variables measured and outcomes calculated** | **Source of sales data** | **Source of composition data** | **Summary of key findings** |
| --- | --- | --- | --- | --- | --- | --- | --- | --- | --- | --- | --- |
| **Cross-sectional studies** | | | | | | | | | | | |
| Alexander et al. 2011 | Major multinational food and beverage companies and informal sector contributions to global food consumption: implications for nutrition policy | To define the contribution of selected players in the food and beverage industry to the sales of packaged foods and soft drinks sold globally and in selected countries  To describe food and soft drink company pledges relate to health, with a focus on IFBA commitments | Global | Company-level data | Cross-sectional | Non-intervention | Food purchases | Top 10 packaged food company shares (% retail value) by country  Top 10 soft drink company shares (% retail value) by country | Euromonitor | N/A | Globally, the top 10 packaged food companies represent 15.2% of sales, with no single company accounting for more than 3.3%. The top 10 soft drink companies represent 52.3% of sales globally. Without participation from small and medium sized businesses, the impacts of health pledges from IFBA will be limited. |
| Baker and Friel 2016 | Food systems transformations, ultra-processed food markets and the nutrition transition in Asia | To measure the extent of market transnationalism market concentration by:  - describing recent trends in ultra-processed food sales, changes in grocery retail distribution channels and food services sales  - Identifying the main transnational food and beverage companies operating in Asia | 12 countries, Asia | Company and product level data | Cross-sectional | Non-intervention | Food purchases | Per capita consumption of ready meals, sweet and savoury snacks, confectionery, biscuits, oils & fats and carbonated soft drinks  Spend and market share of top 10 food and beverage companies  Market concentration in ultra-processed food categories 2004 vs 2013  Distribution share (%) of processed foods through grocery retail channels 1999-2013 | Euromonitor | N/A | The sales of ultra-processed foods have grown rapidly in middle-income countries, with supermarkets and hypermarkets becoming the main channel of distribution. These market forces are likely to be strong drivers of the nutrition transition in Asia. |
| Basu et al. 2013 | Relationship of soft drink consumption to global overweight, obesity and diabetes: a cross-national analysis of 75 countries | To estimate the relationship between soft drink consumption and obesity and diabetes worldwide | 75 countries | Overweight and diabetic population, 1997-2010 | Cross-sectional | Non-intervention | Food purchases | Obesity prevalence  Diabetes prevalence  Per capita consumption of soft drinks | Euromonitor | N/A | A 1% rise in soft drink consumption was associated with an additional 4.8 overweight adults per 100, 2.3 obese adults and 0.3 adults with diabetes |
| Binkley and Golub 2007 | Comparison of grocery purchase patterns of diet soda buyers to those of regular soda buyers | To compare the grocery purchase patterns of regular and diet soda drinkers | USA | 1574 single-purchase households, 1999 | Cross-sectional | Non-intervention | Food purchases | Spending by consumers on different soda types  Volume consumption of soda by type  Demographic characteristics by soda buyer type  Mean annual ounces of food groups purchased by regular soda buyer  Difference in mean annual ounces of food groups purchased by soda buyers | Nielsen | USDA Nutrition Database | Diet soda consumers spend a smaller proportion of their total annual grocery spend on foods higher in calories, such as fruit juice and dairy, and spend a higher proportion on lower fat version of foods, such as dairy products, frozen entrees and salad dressings |
| Bonnet et al. 2014 | Household food consumption, individual caloric intake and obesity in France | To provide a descriptive analysis of individual food consumptions according to household demographics | France | 8,000 households, 2073 products, 2001-2002 | Cross-sectional | Non-intervention | Food purchases | Consumption of energy and macronutrients, per capita per day, BMI classification (normal, overweight, obese)  Share of energy from each macronutrient  Energy and share of energy from macronutrients by age, sex and BMI classification | Kantar | Multiple sources including individual company websites | Results suggest that the consumption of fat and protein is higher than recommended and carbohydrates is lower in those overweight and individuals  Overweight and obese individuals consume more calories at all ages and 20% more fat than those individuals with a normal weight |
| Castetbon et al. 2012 | Purchases of ready-to-eat cereals vary across US household sociodemographic categories according to nutritional value and advertising targets | To describe the ready-to-eat (RTE) cereal purchases according to marketing strategy, nutritional quality and household demographics | USA | 57,171 households that purchased RTE cereals in 2008 | Cross-sectional | Non-intervention | Food purchases | Total spend on 249 individual products, the total number of households that purchased each product and their frequency of purchase  The nutrition composition of each product  The targeted advertising exposure (“child-targeted”, “adult-targeted” or “family-targeted”) | Nielsen | Cereal FACTS (Food Advertising to Children and Teens Score) report, Rudd Centre for Food Policy and Obesity, 2009 | African-American and Asian households, those earning less than US$30,000 per annum and those with one or more children purchased the most RTE cereal  The nutritional quality of cereal purchases was lower in African-American and Hispanic households |
| Combris et al. 2011 | Improvement of the nutritional quality of foods as a public health tool | To assess the potential contribution of improving the nutritional quality of breakfast cereals, bread-based products and biscuits/pastries on individual nutrition intake  To discuss the means to encourage food manufacturers to implement reformulation | France | 20,000 households, 2006 | Cross-sectional | Non-intervention | Nutrition composition | Nutrient content of products (sugars, fat, fibre and sodium g/100g)  Total quantity of sugars, fat, fibre and sodium delivered to the market | Kantar | Oqali database (FULL REF) | Improvement in the sugar, fat, fibre and salt content in the products with the lowest nutritional quality would lead to significant variation in an individual’s nutrient consumption  Encouraging reformulation is a worthy target for policy makers |
| De Roos et al. 2016 | Differences in expenditure and amounts of fresh foods, fruits and vegetables, and fish purchased in urban and rural Scotland | To quantify expenditure on all fresh foods, fruits, vegetables and fish across urban and rural households in Scotland | Urban and rural Scotland, UK | 2567 urban and rural households | Cross-sectional | Non-intervention | Food purchases | Mean weekly expenditure (£/adult equivalent) on fresh food, F&V and fish  Mean weekly amount (kg/adult equivalent)  Average prices per kg by urban and rural areas | Kantar | N/A | Rural households had the highest level of spending on fresh foods and F&V and also bought the most volume (kg) of these items.  Prices paid for products was higher in urban compared to rural areas.  These data suggest that the higher expenditure in rural areas is due to factors other than pricing and availability of products. |
| Eyles et al. 2016 | Estimating population food and nutrient exposure: a comparison of store survey data with household panel food purchases | Compare estimates of nutrient exposure from a packaged food store survey with those from a household panel | New Zealand | 16,812 products from 1229 households, compared to 8440 products, 2012 | Cross-sectional | Non-intervention | Nutrition composition | Mean content of saturated fat, sodium, sugar and energy content of packaged food products  Mean difference between nutrient content of household panel products and survey products | Nielsen | Nutritrack database | Products recorded from the store survey had a higher sodium and sugar content compared to those from the household panel |
| **Author-date** | **Title** | **Objective** | **Geography** | **Demographic** | **Study design** | **Intervention or non-intervention** | **Measuring patterns in food purchases or nutrient content of foods** | **Variables measured and outcomes calculated** | **Source of sales data** | **Source of composition data** | **Summary of key findings** |
| Jones et al. 2017 | An evaluation of the healthiness of the Indian packaged food and beverage supply | To use nutrient profiling to examine the healthiness of packaged food products from India’s top manufacturers | India | N/A | Cross-sectional | Non-intervention | Food purchases | Sales-weighted health star rating (HSR) of food and beverage products  % sales of products with HSR >3.5 (“healthy”) by company  Mean HSR by company and product category | Euromonitor | FoodSwitch  Data direct from manufacturer | Healthiness of Indian packaged food was low, with an overall mean HSR 1.8 out of 5 and only 17% of products classified as ‘healthy’.  There was significant variation between companies (0.5-3.0) based on the types of product and the nutritional composition of individual products |
| Juhl and Jensen 2014 | Relative price changes as a tool to stimulate more healthy food choices – A Danish household panel study | To examine the effects of discounting on the purchasing of low-fat and full-fat yoghurt | Denmark | 2000 households that purchased yoghurt, 2010-2011 | Cross-sectional | Non-intervention | Food purchases | Number of full-fat and low-fat products purchased  Listed prices  Levels of discount applied  Household income  Number of children present in household | GfK  Mintel | N/A | Discounting has an asymmetric effect on full-fat and low-fat yoghurts, with discounting |
| Mhurchu et al. 2011 | Sodium content of processed foods in the United Kingdom: analysis of 44,000 foods purchased by 21,000 households | To determine if household panel data can be used to assess the sodium content of processed foods  To estimate the mean sodium content of processed foods | Great Britain | 44,372 foods purchased in 21,108 households, 2008-2009 | Cross-sectional | Non-intervention | Nutrition composition | Product description product weight, number of annual purchases  Sodium content (mg/100g)  Unweighted and sales-weighted mean sodium values calculated | Kantar | Kantar | Table salt, processed meat and bakery products were the largest contributors to sodium purchases. For some products, including bread, cereals and processed meats, sales-weighted sodium means were higher than unweighted means, suggesting that market-leading products contain more salt. Targeting a small number of product categories for reformulation could have a significant public health benefit. |
| Nakumura et al. 2015 | Price promotions on healthier compared with less healthy foods: a hierarchical regression analysis of the impact on sales and social patterning of responses to promotions in Great Britain. | To establish if less-healthy foods are more likely to be promoted than healthier food  To find out if consumers are more responsive on less-healthy products  To establish if there are socioeconomic differences in food purchases in response to price promotions | UK | 11,323 products in 26,986 households, 2010 | Cross-sectional | Non-intervention | Nutrition composition | Household demographics, including age, sex, income, educational attainment and self-reported BMI  Total number of units of each product sold in each household per week  Price and promotional status of each purchase used to calculate total number of promotional purchases per week  Nutrient profile score of each product | Kantar | Kantar | 60% of products analysed were healthy, 40% were less healthy. A one-point increase in the category mean nutrient profile score was associated with a 7.7% increase in sales (the higher the NP score, the less the product). The uplift in sales of promotional items was higher in low SES households compared to high SES households, although there was no significant SES gap in the purchase of less-healthy foods on promotion. |
| Pechey and Monsivais 2015 | Supermarket Choice, Shopping Behavior, Socioeconomic Status, and Food Purchases. | To assess the contributions of supermarket choice and shopping behaviours to the healthfulness of purchases and social patterning in purchases | UK | 24,879 households stratified by social class, 2010 | Cross-sectional | Non-intervention | Food purchases | Household demographics, including occupation, income, age  Households classified by social class  Number of trips to different types of supermarkets per month  Proportion of ‘small trips’ to supermarkets (10 items or fewer)  Mean number of different chains visited per month  Whether households used a primary chain  Proportion of energy from less-healthy foods and soft drinks  Proportion of energy consumed from fruits and vegetables | Kantar | Kantar | Households using low-price supermarket chains to shop purchased significantly less energy from fruits and vegetables and a higher percentage from less-healthy foods and beverages. More frequent trips and fewer small trips were associated with healthier purchasing choices. Although supermarket choice and shopping behaviour are both associated with the healthfulness of purchases, neither appears to contribute to socioeconomic differences |
| Pechey and Monsivais 2016 | Socioeconomic inequalities in the healthiness of food choices: Exploring the contributions of food expenditures | To explore the extent to which food expenditure mediates socioeconomic inequalities in the healthiness of household food choices | UK | 24,879 households stratified by social class, 2010 | Cross-sectional | Non-intervention | Food purchases | Head of household occupation as indicator of socioeconomic status  Total household spend on take-home food and beverages over 52-week period of 2010  Energy adjusted food expenditure (£ per 200 kcal)  Supermarket choice  Healthiness of food and beverage choices | Kantar | Kantar | Higher social class was associated with higher expenditure on food and healthier purchases, suggesting that lower expenditure on food leads to less-healthy food choices for low socioeconomic groups. |
| Slining et al. 2015 | An approach to monitor food and nutrition from factory to fork | To develop an approach that links purchase and consumption data of food and beverage products | USA | 38,311 uniquely formulated foods, 2007-2008 | Cross-sectional | Non-intervention | Nutrition composition | Mean daily intakes of energy, total sugars, saturated fat and sodium | Nielsen | USDA National Nutrient Database  Mintel  Datamonitor | Depending on the source of the nutrition information, differences in the daily calories, sugars and sodium was observed. By combining commercial food and beverage purchases with national nutrition databases, changes in the US food supply can be captured |
| Stern et al. 2016 (2) | Where people shop is not associated with the nutrient quality of packaged foods for any racial-ethnic group in the United States. | To examine whether the mix of food stores people shop at is associated with the nutrient profile of packaged food purchases | USA | 368,934 household-year observations, 2007-2012 | Cross-sectional | Non-intervention | Nutrition composition | Household demographics, including ethnicity, age, educational attainment and income  Name and store type of every purchase  Nutrition composition of foods  Categorisation of households into three groups: primarily grocery cluster, primary mass-merchandiser cluster and combination cluster | Nielsen | Gladson Nutrition Database | Shopping primarily at grocery stores was not associated with a better nutrient profile of household PFPs compared to the mass-merchandisers or combination clusters. These results were consistent across ethnic groups. Policies that promote access to different types of stores may be ineffective as a public health intervention. |
| Stewart et al. 2011 | Can low-income Americans afford to satisfy MyPyramid fruit and vegetable guidelines? | To estimate the cost of meeting the My Pyramid fruit and vegetable guideline | USA | 64,440 households, 2008 | Cross-sectional | Non-intervention | Food purchases | Total expenditure on foods and volumes purchased  Calculated price per ounce/pint  Estimated cost-per-cup equivalent | Nielsen | N/A | In 2008, a wide variety of fruits and vegetables cost US$0.40-0.50 per cup equivalent. If households spend 40-50% of their food budget on fruits and vegetables, they should be able to satisfy the MyPyramid guideline |
| **Author-date** | **Title** | **Objective** | **Geography** | **Demographic** | **Study design** | **Intervention or non-intervention** | **Measuring patterns in food purchases or nutrient content of foods** | **Variables measured and outcomes calculated** | **Source of sales data** | **Source of composition data** | **Summary of key findings** |
| Thunstrom 2010 | Preference Heterogeneity and Habit Persistence: The Case of Breakfast Cereal Consumption | To examine the strength and heterogeneity in the habit persistence of breakfast cereals | Denmark | 181 breakfast cereals purchased by 926 households, 2003, mixed multinomial logit model | Cross-sectional | Non-intervention | Food purchases | Household demographics, including income, educational attainment and family size  Price of products (SEK per 100g)  Grams of fat, saturated fat, fibre, sugar and salt per 100g of product  Presence of Keyhole label | GfK | Swedish National Food Association (SLV) | Breakfast cereal purchase is highly habitual, with the degree of habit persistence varying across households, with it being significantly weaker for households with several adults or children compared to one-adult households |
| Whybrow et al. 2017 | Dietary patterns of households in Scotland: Differences by level of deprivation and associations with dietary goals. | To evaluate dietary patterns from food and drink purchase data from Scotland and to identify dietary patterns that were associated with adherence to the Revised Scottish Dietary Goals (RSDG) | Scotland, UK | 720 households, 2010 | Cross-sectional | Non-intervention | Food purchases | Household size and composition  Scottish Index of Multiple Deprivation  Weights of food and beverage products purchased  Weights adjusted for avoidable and unavoidable food waste  Nutrition composition of products  Energy density (kcal/100g) of foods  Dietary Quality Index of each food | Kantar | Kantar | The mean DQI score for all food products was 38 (out of 100), indicating few dietary goals were being met. Six dietary patterns were identified, accounting for 35% of total variance in food intake. Three dietary patterns were associated with significantly lower DQI score (less healthy diet) and one significantly higher (more healthy diet). |
| Whybrow et al. 2017a | Social deprivation is associated with poorer adherence to healthy eating dietary goals: analysis of household food purchases | To estimate dietary intake relevant to the SDGs based on levels of household deprivation | Scotland, UK | 2586 households, 2012 | Cross-sectional | Non-intervention | Food purchases | Household size, composition and income  Total weight of products purchased  Nutrition composition of products  Equalised per person intakes of fruits and vegetables, red and processed meat, oily fish, fibre  Percentage energy from fat, saturated fat and non-milk extrinsic sugars  Energy density of foods (kcal/100g) | Kantar | Kantar | As deprivation increased, households were further away from achieving the SDGs. Intakes of oily fish and F&V decreased ,and intakes of red and processed meat increased as deprivation increased. |
| Yan et al. 2016 | Asymmetric demand patterns for products with added nutritional benefits and products without nutritional benefits | To investigate consumer demand patterns for products with and without nutritional benefits across processed healthy and unhealthy foods | UK | 6218 households, 800,000 transactions | Cross-sectional | Non-intervention | Food purchases | Descriptive statistics of households, including age, ethnicity, education and income  Healthiness of each food transaction  Nutritional benefits of product purchased, taken from label (e.g. reduced fat, low salt)  Price of products purchased and price changes | Kantar | N/A | Consumers are more sensitive to price decreases and less sensitive to price increases for both healthy and unhealthy food  These sensitivities are greater for products with no nutritional benefit, supporting the hypothesis that products with nutritional benefits have a higher brand equity than those without |
| **Longitudinal studies** | | | | | | | | | | | |
| Anders and Moser 2010 | Consumer choice and health: The importance of health attributes for retail meat demands in Canada | To estimate health behaviour and purchase patterns for fat-reduced ground meats that are believed to be “good-for-you” compared to regular products | Canada | 9,000 households, 220,000 individual purchases | Longitudinal | Non-intervention | Food purchases | Mean number of purchases and mean expenditure of each purchase, by meat type  Retail prices ($/kg) and quantity (tonnes) by generic and branded products  Price elasticities by product type | Nielsen | N/A | When it comes to meat purchased at retail, preferences for health triumph over those for taste.  “Extra-lean” ground meats have become a staple of the Canadian diet.  These results are a starting point for a more in-depth understanding of health behaviours |
| **Author-date** | **Title** | **Objective** | **Geography** | **Demographic** | **Study design** | **Intervention or non-intervention** | **Measuring patterns in food purchases or nutrient content of foods** | **Variables measured and outcomes calculated** | **Source of sales data** | **Source of composition data** | **Summary of key findings** |
| Andersen and Smed 2013 | What is it consumers really want, and how can their preferences be influenced | To investigate whether price changes or information would be the best policy instrument to reduce the consumption of fat from milk | Denmark | 1700 households over 1997-2004 | Longitudinal | Non-intervention | Food purchases | Volume sales of milk by fat content type and demographic factors  Price of product by region, store type, mode of production (organic vs conventional)  Preferred fat share (g/L) by household demographics  Reaction to information campaign based on changes in optimum fat share  Estimated price elasticity for fat | GfK | N/A | Consumer who purchase low fat milk purchase a higher volume than those who buy milk with a higher fat content  Consumers who prefer high fat milk do not react to information about its health effects but do react to prices  Consumers who prefer low fat milk are influenced by information but are not as price sensitive |
| Batis et al. 2016 | First-Year Evaluation of Mexico’s Tax on Nonessential Energy-Dense Foods: An Observational Study | To examine changes in volume of taxed and untaxed packaged food purchases in response to Mexico’s tax on energy-dense foods, by socioeconomic status and for the entire sample | Urban Mexico | 6248 households, 2012-2014 | Longitudinal | Intervention | Food purchases | Socioeconomic status (SES) score  Consumption of food categories in grams per capita per month | Nielsen | N/A | The mean volume of purchases of taxed food products declined. Low SES households purchased 10.2% less taxed foods than expected, medium SES households 5.8% and no change in high SES households. |
| Berning 2014 | The effects of breakfast cereal coupons on the nutritional quality of household purchases | To examine what effect coupon use has on the nutritional quality of consumer purchases of breakfast cereals | New York City, USA | 1442 households that made breakfast cereal purchases in 2006, 2007, 2008 | Longitudinal | Intervention | Food purchases | Nutritional composition of breakfast cereals purchased with coupons and without.  Purchases by household demographics and coupon use.  % change in fat, fibre, protein, sodium and sugar content per serving between coupon types | Nielsen | USDA National Nutrition Database  Nutribase 9 software  Health Canada | Breakfast cereals purchased using a manufacturer or retailer coupon contain more sugar and salt than those purchased without a coupon. |
| Caillavet et al. 2018 | Is nutritional quality of food-at-home purchases improving? 1969-2010: 40 years of household consumption surveys in France. | To observe changes in food-at-home purchases by French households and their impacts in their nutritional quality over 1969-2010 | France | 5,000-20,000 households depending on year | Longitudinal | Non-intervention | Food purchases | Energy content of food purchases (kcal per capita per day) by year  Purchases of meat and dairy products, fat, fruits and vegetables, cereal-based products and sugar-based products (g/200kcal) by year  Energy contributions of macronutrients (%) | Kantar | CIQUAL food composition table | Overall, there was an increased trend towards processed foods compared to raw products in 2010 vs 1989  The purchase of calories increased by 6.7% and the energy density of products improved  However, in the last decade, while nutrient density has ceased to increase, the overall purchase of has increased. |
| Cho et al. 2011 | An analysis of the Olympic sponsorship effect on consumer brand choice in the carbonated Soft drink market using household Scanner data | To investigate the effects of Coca-Cola’s sponsorship on consumers soft drink choices during the 2006 Winter and 2008 Summer Olympic Games | USA | 11,887 households | Longitudinal | Intervention | Food purchases | Consumer expenditure ($) and market share (%) of top cola brands (Coca-Cola and Pepsi)  Quantities purchased and advertising expenditures of Coca-Cola and Pepsi | Nielsen | N/A | Olympic sponsorship generated significantly greater consumer choices for Coca Cola over Pepsi during the Games  Results of the model show that advertising effects aside, sponsorship effect on purchasing behaviour exists, although it has short term results |
| Cleeren et al. 2016 | Regular or low-fat? An investigation of the long-run impact of the first low-fat purchase on subsequent purchase volumes and calories | To examine the effectiveness of the first low-fat purchase of chips/crisps on subsequent purchased volume and calories | Netherlands | 311 households that purchased chips/crisps, 2004-2007 | Longitudinal | Non-intervention | Food purchases | Total volume sales of crisps/chips per year  Total number of calories purchased per year  Total number of low-fat products available  Price of products  Advertising  Spending in other food categories | GfK | GfK | The purchase of a low-fat chip/crisp product can lead to the over-purchase of products in the long- and short-term. |
| **Author-date** | **Title** | **Objective** | **Geography** | **Demographic** | **Study design** | **Intervention or non-intervention** | **Measuring patterns in food purchases or nutrient content of foods** | **Variables measured and outcomes calculated** | **Source of sales data** | **Source of composition data** | **Summary of key findings** |
| Colchero et al. 2016 | Beverage purchases from stores in Mexico under the excise tax on sugar sweetened beverages: observational study | To find out what effect the excise tax on sugar sweetened beverages has had on the purchase of beverages from stores in Mexico | Urban Mexico | 6253 households | Longitudinal | Intervention | Food purchases | Descriptive statistics of the households  Absolute and relative differences in expected volume purchased and post-tax volumes purchased  Monthly predicted purchases of taxed beverages by socioeconomic status | Nielsen | N/A | Purchases of taxed beverages declined by an average of 6%, or -12mL per capita per day. There was a reduction in all three socioeconomic groups, although the decline was greatest in the low SES group |
| Edenbrandt and Jansen 2018 | A hedonic analysis of nutrition labels across product types and countries | To increase the understanding of whether consumers value the assistance nutrition labels aim to provide | Netherlands and Denmark | 2,998,073 individual items purchased | Longitudinal | Intervention | Food purchases | Number of products sold  Presence of label/product characteristic: organic, Fulfill choices/Keyhole criteria  Store type  Price  Size | GfK | N/A | Products that fulfil the Choices criteria in the Netherlands are more expensive than those that do not.  In Denmark, products that fulfil the Keyhole criteria and display the label have increased more in price than those that meet the criteria but don’t display the label |
| Eyles et al. 2013 | Impact of the UK voluntary sodium reduction targets on the sodium content of processed foods from 2006 to 2011: Analysis of household consumer panel data | To determine what impact the FSA voluntary sodium reduction targets had on the sodium content of processed foods between 2006 and 2011 | UK | 47,339 products purchased by 18,099 households, 2006-2011 | Longitudinal | Intervention | Food purchases | Household demographics  Crude and sales-weighted sodium content (mg/100g) of processed food products  Mean difference in sodium content (2006 vs. 2011)  Number and percentage of products meeting 2010 sodium target in 2006 vs. 2011 | Kantar | Kantar | Between 2006-2011, there was a mean reduction in sodium content of -26mg/100g, equivalent to a fall of 7%, indicating that the FSA sodium targets have delivered a moderate reduction |
| Ford et al. 2014 | Are food and beverage purchases in households with pre-schoolers changing? A longitudinal analysis from 2000-2011 | To describe changes in consumer packaged goods purchases between 2000 and 2011 among households with children aged 2-5 years | USA | 42,753 households with one or more child aged 2-5 years, 2000-2011 | Longitudinal | Non-intervention | Food purchases | Calories purchased per capita from foods and beverages  Calories purchased per capita by race, female head of household education and household income | Nielsen | Does not specify | Total calories from purchased packaged food and beverages declined significantly over 2000-2011, with milks, juices, grain-based desserts, savoury snacks and sweet snacks and candy among the major changes. Changes varied significantly between race, female head of household education level and household income. |
| Griffith et al. 2015 | Relative prices, consumer preferences, and demand for food | To understand how large changes in relative food prices affect the nutritional quality of households shopping baskets over 2006-2011 | UK | 22,000 households, demand model | Longitudinal | Non-intervention | Food purchases | Change in relative prices by food groups  Mean calorie and macronutrient purchases across time periods  Calories (per person per day) and % of total calorie share by food group | Kantar | Kantar | Changes in the relative prices of foods over time seem to have a negative effect on the composition of foods purchased  While relative prices undoubtedly affect food purchases, failure to model other factors, such as consumer preferences, can lead to the misestimation of the impact of price on nutrition |
| Grummon and Taillie 2017 | Nutritional profile of supplemental nutritional assistance program household food and beverage purchases | To describe the usual purchases of SNAP households, income-eligible households and higher-income households across key food, beverage and nutrient groups. | USA | 70,447 households split by SNAP participant, income-eligible non-participant and higher income households, 2012-2013 | Longitudinal | Intervention | Food purchases | Store purchases of food and beverage groups in nutrient (g/kcal)/person/day split by social demographics  Household size, number and age of children, marital status, age of household head, race/ethnicity, maximum educational attainment, income | Nielsen | Doesn’t specify source – linked by unique product code (UPC) to nutrition facts panel | SNAP households purchased on average 565 kcal/person/day from SSBs and junk food. Average household purchases did not meet dietary guidelines for fruits, vegetables, saturated fat, fibre or sodium. Compared to non-participants, SNAP households purchased 15-20 kcal more from SSBs, more total calories and more total sodium. |
| Jensen and Smed 2013 | The Danish tax on saturated fat - Short run effects on consumption, substitution patterns and consumer prices of fats | To assess the market effects of the Danish saturated fat tax on butter, butter blends, margarines and oils | Denmark | 2000 households, 2008-2012 | Longitudinal | Intervention | Food purchases | Descriptive statistics of panel members, including residence, further education levels and family composition  Annual consumption of butter, butter blends, margarines and oils in kg/individual  Market share of discount stores (% volume of product purchases)  Average saturated ƒat content (g/100g)  Price (DKK/kg) | GfK | Dos not specify source of average saturated fat content (g/100g) | The introduction of a saturated fat tax on the considered products reduced consumption by 10-15%. Results also suggest that there was a shift away from purchases in high-end retailers to discounter stores. Data also suggest that some retailers increased the price of products more than the pure tax increase. |
| Levy and Shrapnel 2014 | Quenching Australia’s thirst: A trend analysis of water-based beverage sales from 1997-2011 | To show trends in sugar sales of sugar-sweetened and non-sugar sweetened water-based beverages in Australia over a 15-year period | Australia | 10,000 households, 2007-2011 | Longitudinal | Non-intervention | Food purchases | Volume sales, annual grown rate, difference per capita and losses and gains of volume share by beverage category and year  Sugar contribution in tonnes and kg per person by year and beverage category  Demographic penetration of beverage categories (% persons consuming) | Nielsen | N/A – rounded estimates by beverage category only | Sales of water-based beverages grew by 1.7% per annum, driven mainly by the growth of non-sugar varieties (4.9% per annum)  Volume share of non-sugar varieties increased from 30% to 42% over the 15-year period  Sugar contributions from water-based beverages fell from 9.2kg to 7.6kg per person  Soft drinks, energy drinks and sports drinks were most popular with teenagers and young adults |
| Lichtman-Sadot 2016 | Does banning carbonated beverages in schools decrease student consumption? | Do address what impact banning beverage consumption at schools has on household compensation | USA | 7517 households residing in selected school districts, 2004-2009 | Longitudinal | Intervention | Food purchases | Demographics of household, including number of children, ethnicity and income  Details of local school soda policy (type of soda ban, date implemented)  Monthly soda and diet-soda consumption by household | Nielsen | N/A | When high-schools ban the sale of carbonated beverages to students, households with an affected student increase their consumption of non-diet sodas by 3.4 cans per month, offsetting the impact of the high-school ban |
| Lopez-Olmedo et al. 2018 | The Socioeconomic Disparities in Intakes and Purchases of Less-Healthy Foods and Beverages Have Changed over Time in Urban Mexico | To examine the relationship between socioeconomic status (SES) by examining household purchase and individual food intake data | Urban Mexico | 5240 households stratified by SES, 2012-2014 | Longitudinal | Non-intervention | Food purchases | Demographics of household, including number of occupants, their ages, educational attainment and the number of rooms and cars  Per capita daily purchases of healthy and less healthy foods by household SES (g/day) | Nielsen | N/A | Higher SES households purchased more less-healthy foods and more healthy beverages and lower SES households had purchased more less healthy beverages. This group also saw the largest reduction in these purchases from 2012-2014. |
| Mathias et al. 2015 | Monitoring changes in the nutritional content of ready-to-eat grain-based dessert products manufactured and purchased between 2005 and 2012. | To examine the changes in the nutritional content of grain-based ready-to-eat desserts (GBDs) | USA | 134,128 households that purchased grain-based desserts from 2005-2012 | Longitudinal | Non-intervention | Nutrition composition | Demographics of household, including income, ethnicity, education levels and family composition  Number of GBDs that carry a nutrition facts panel  Energy, saturated fat and sugar density by year  GBD purchases (g/person/day) | Nielsen | Unspecified commercial database of nutrition facts panels | The saturated fat content of GBDs increased the sugar content decreased from 2005 to 2012, resulting in significant reductions in energy density (433 kcal/100g to 422 kcal/100g). Household purchases of GBD fell by 24%. These results highlight the opportunity for manufacturers and public health officials to develop reformulation strategies. |
| Mendez Lopez et al. 2017 | Is trade liberalisation a vector for the spread of sugar-sweetened beverages? A cross-national longitudinal analysis of 44 low- and middle-income countries. | To test whether lower tariffs effectively increase exports of sugar sweetened beverages (SSBs) | 44 LMICs | Company and product level sales data | Longitudinal | Non-intervention | Food purchases | Sales of SSBs by country in per capita per litre  Import value (US$) of SSBs per annum  Population size used to calculate imports of SSBs per capita | Euromonitor | N/A | For every 1% increase in tariffs in LMICs, import of SSBs fell by 2.9%. In turn, increased imports of SSBs were associated with an increase in SSB consumption, with a 10% increase in SSB imports resulting in an increased consumption by 0.36L per person. |
| Mhurchu et al. 2017 | Effects of a Voluntary Front-of-Pack Nutrition Labelling System on Packaged Food Reformulation: The Health Star Rating System in New Zealand. | To evaluate the effects of the Health Start Rating (HSR) on food reformulation | New Zealand | 35,602 products purchased by 1726 households, 2014-2016 | Longitudinal | Intervention | Food purchases | Numbers of products displaying HSR labels  Nutritional composition of products  Number of units purchased  Mean changes in nutrient composition over time | Nielsen | Nutritrack database | In 2016, 5.3% of products carried a HSR label, with the highest rates among cereals and convenience foods. Products displaying an HSR label had higher energy, fibre and sodium densities compared to non-HSR products. Reformulation of HSR products was greater over the same time period compared to non-HSR products, suggesting that the adoption of the HSR system is driving the healthier reformulation of some products |
| **Author-date** | **Title** | **Objective** | **Geography** | **Demographic** | **Study design** | **Intervention or non-intervention** | **Measuring patterns in food purchases or nutrient content of foods** | **Variables measured and outcomes calculated** | **Source of sales data** | **Source of composition data** | **Summary of key findings** |
| Ng et al. 2012 | Use of caloric and non-caloric sweeteners in US consumer packaged foods, 2005-2009 | To examine the extent of caloric sweetener and non-caloric sweetener use in the consumer packaged foods and beverages purchased by US households | USA | 85,451 uniquely formulated foods | Longitudinal | Non-intervention | Nutrition composition | Percentage of packaged foods purchased by type of sweetener  Most common sweeteners used in packaged foods  Percentage of foods containing caloric and non-caloric sweeteners | Nielsen | Gladson Nutrition Database | Of the 85,451 products examined, 75% contain a sweetener (68% caloric sweetener, 1% non-caloric and 6% both). 77% of all calories purchased contained caloric sweeteners and 73% of food volume contains caloric sweeteners. These results highlight the challenge in monitoring sweetener consumption. |
| Ng et al. 2014 | Turning point for US diets? Recessionary effects or behavioural shifts in foods purchased and consumed | To examine if declining energy intakes suggest a shift in dietary patterns that are independent of a turbulent economic period | USA | 108, 932 individual households, 2000-2011 | Longitudinal | Non-intervention | Food purchases | Average calories purchased per capita per day split by foods, beverages and total by year  Average calories purchased per capita per day by ethnicity and employment status | Nielsen | Mintel GNPD | Children were the group that decreased their calorie intake the most  After adjusting for economic factors, caloric purchases fell significantly from 2003-2011.  The period of the great recession was associated with a small increase in caloric purchases, with a 1% increase in the levels of unemployment associated with a 1.6-4.1 kcal per capita per day increase in the total calories purchased. |
| Ng et al. 2016 | Trends in racial/ethnic and income disparities in foods and beverages consumed and purchased from stores among US households with children, 2000–2013 | To determine if there are disparities in calories obtained from packaged foods between different ethnic and income groups | USA | 64,709 households | Longitudinal | Non-intervention | Food purchases | Energy intake, kcal per capita, by store and non-store sources and ethnic group  Energy intake, kcal per capita, by store and non-store sources and income group  % food calories from packaged foods manufactured by healthy weight commitment foundation company, no-NWCP company or private label company | Nielsen | Nutrition facts panel, various sources | Store-bought foods represent the largest component of dietary intake  Calories purchased from non-store source foods and store sourced beverages declined the most over 2000-2013  A slower reduction was seen amongst non-Hispanic black and low income households compared to non-Hispanic white and high income households |
| Ng et al. 2018 | Federal nutrition program revisions impact low-income households’ food purchases | To examine changes in household food purchases across the US from 2008-2014 among WIC participating and non-participating households | USA | 65,000 households | Longitudinal | Intervention | Food purchases | Product sales volumes and nutrient contents expressed as energy (kcal), total sugars, fats, sodium and fibre (g) purchased per capita per day  Classification of food products into “WIC eligible” and “WIC ineligible” categories  Classification of food products by degree of industrial food processing and convenience of preparation | Nielsen | Gladson Nutrition Database  Mintel  Datamonitor | WIC-eligible households saw a significant decreases in the purchase of calories, sodium, fat and sugar, as well as reductions in grains, gran-based desserts, higher-fat milk and SSBs.  There were increased purchases of fruit and vegetables with no added sugar/salt.  Income eligible non-participating households had similar but smaller reductions  WIC packaged revisions seem to have had a positive impact on the nutritional profile of food purchases |
| Oh et al. 2016 | Did Revisions to the WIC Program Affect Household Expenditures on Whole Grains? | To examine the purchases of whole grain products before and after the 2009 changes to the Supplemental Nutrition Program for Women, Infants and Children (WIC) | USA | 36,477 households reporting grain purchases, 2008-2010 | Longitudinal | Intervention | Food purchases | Household demographics, including income, educational attainment, size and age  Monthly wholegrain expenditures (US$)  Monthly refined grain expenditures (US$)  Monthly whole grain amount (ounces)  Monthly refined grain amount (ounces) | Nielsen | N/A | Participation in the WIC program was associated with more wholegrain purchases over 2008-2010. The changes approximately doubled the associated effect of WIC participation on the purchases of wholegrain products. The results confirm the recommendations that changing assistance programs can lead to positive effects on dietary choices. |
| Piernas et al. 2015 | A Dynamic Panel Model of the Associations of Sweetened Beverage Purchases With Dietary Quality and Food-Purchasing Patterns | To examine the independent associations between beverages sweetened with caloric and low-calorie sweeteners (CS and LCs) with dietary quality and food purchasing patterns | USA | 34,294 households, 2000-2010, dynamic panel model | Longitudinal | Non-intervention | Food purchases | Household demographics, including age, sex, educational attainment, income and ethnicity  Total energy from all purchases  Total energy excluding CS and LCS  Total energy from foods  Total energy from macronutrients  Servings per day of LCS and CS beverages (servings/day) | Nielsen | Gladson Nutrition Database | Each 1 serving/day increase in consumption of either CS or LCS beverages resulted in increased purchases of total calories, carbohydrates, total sugars and total fats. Consumers of CS and LCS beverages had a poorer dietary quality overall. |
| Poti et al. 2015 | Is the degree of food processing and convenience linked with the nutritional quality of foods purchased by US households? | To define categories for classifying products by the degree of food processing and convenience  To determine the trends in the caloric contribution of processed and convenience foods  To compare saturated fat, sugar and sodium content of packaged food purchases across all levels of processing and inconvenience | USA | 157,142 households, 2000-2012 | Longitudinal | Non-intervention | Nutrition composition | Proportion of total calories purchased by different levels of food processing and convenience  Mean nutrient content of foods by levels of processing and convenience  Proportions of households whose purchases exceed dietary guidelines on saturated fat, sugar and sodium as defined by degree of processing and convenience | Nielsen | Mintel GNPD | Three quarters of energy in food purchases came from moderately and highly processed foods and beverages in 2012, with no significant change from 2000. When classifying foods by convenience, ready-to-eat and ready-to-heat products formed most of the energy purchased. |
| Poti et al. 2016 | Highly Processed and Ready-to-Eat Packaged Food and Beverage Purchases Differ by Race/Ethnicity among US Households | To examine the association between ethnicity/race with highly processed and ready to eat food purchases | USA | 1.2 million individual food products purchased by 157,142 households, 2000-2012, regression model | Longitudinal | Non-intervention | Food purchases | Household demographics, including age, education, income and ethnicity  Proportion of saturated fat (% kcal), sugar (%kcal) and energy density (kcal/10  0g food) of packaged food products | Nielsen | Gladson Nutrition Database | Black households had lower purchases of highly processed foods and RTE convenience foods and higher purchases of basic processed foods such as oils and sugars, when compared to white households. Black households purchased foods that were more energy dense and had higher median sugar content. Highly processed convenience foods are associated with, but cannot fully explain, ethnic disparities in the nutritional quality of processed foods |
| **Author-date** | **Title** | **Objective** | **Geography** | **Demographic** | **Study design** | **Intervention or non-intervention** | **Measuring patterns in food purchases or nutrient content of foods** | **Variables measured and outcomes calculated** | **Source of sales data** | **Source of composition data** | **Summary of key findings** |
| Poti et al. 2017 | Sodium Reduction in US Households' Packaged Food and Beverage Purchases, 2000 to 2014. | To assess the change in the amount of sodium acquired from packaged foods, the sodium content of these products and the proportion of households that purchase products with an optimum sodium density | USA | 1.5 million individual food products purchased by 172,042 households, 2000-2012 | Longitudinal | Non-intervention | Nutrition composition | The amount of sodium purchased daily per person (mg/person/day)  Sodium content of food products (mg/100g)  Sodium density of food products (mg/100kcal) | Nielsen | Gladson Nutrition Database | The amount of sodium purchased from packaged foods fell by 396mg/day per capita from 2000 to 2012. The sodium content of products fell by 12%, however in 2012 only 2% of households had packaged food purchases with a sodium density of 1.1mg/kcal or less. Despite signs that sodium purchases have fallen, most households are still buying products with excessive sodium densities. |
| Slining et al. 2013 | Food companies’ calorie-reduction pledges to improve US diet | To identify the food categories that contributed to total number of calories purchased and relate these to the pledges made by the companies of the Healthy Weight Commitment Foundation (HWCF) | USA | 50,000 households over 2000-2015 | Longitudinal | Intervention | Food purchases | Average calories purchased from packaged good foods, split by HWCF and non-HWCF brands, ethnicity, age and income | Nielsen | Gladson Nutrition Database | HWCF companies accounted for 25% of calories consumed in the US in 2007, and that the calorie reduction pledge of 1.5 trillion calories accounts for 0.8% of total calories sold across all consumer packaged foods and beverages.  The effects pledges have on foods supply and individual diets is important to developing new policies to improve nutrition. |
| Smed et al. 2016 | The effect of Danish saturated fat on food and nutrient intake and modelled health outcomes: an econometric and comparative risk assessment evaluation | To evaluate the effect of the Danish fat tax on saturated fat in terms of saturated fat, unsaturated fat, salt, salt, fruit and vegetable consumption and to model the associated changes in mortality | Denmark | 2500 households | Longitudinal | Intervention | Food purchases | Sociodemographics of the panel (age, residence, education, family composition)  % changes in consumption compared with baseline (taken from national dietary survey) by age and sex  Deaths averted and delayed by cause | GfK | Danish Food Composition Databank | The introduction of the saturated fat tax in Denmark had a minor but positive impact on public health.  It led to a 4.0% reduction in the consumption of saturated fat.  Vegetable and salt consumption increased as a result.  A modelled 123 lives were saved annually, equal to 0.4% of all deaths from NCDs in Denmark |
| Smed et al. 2017 | The consequences of unemployment on diet composition and purchase behaviour: a longitudinal study from Denmark | To explore and describe the effect unemployment has on food purchase behaviour and composition of diets | Denmark | 3440 households, 2008-2012 | Longitudinal | Non-intervention | Food purchases | Nutrient composition per 100g of foods purchased  Type of store frequented  Monthly purchases of food products  Total food expenditure  Unemployment status of household, including welfare benefit status, unemployment benefit or supported employment | GfK | Food Composition Databank, National Food Institute, Technical University of Denmark | In the short-run, unemployment led to substitution of discount store products and higher levels of expenditure on products high in fats and protein, mainly due to increased purchasing of animal products. Medium-term, unemployment led to a reduction in total food expenditure, along with a decline in the purchase of animal products, fats and protein, indicating that unemployment has a significant impact on diet composition |
| Spiteri and Soler 2017 | Food reformulation and nutritional quality of food consumption: an analysis based on households panel data in France. | To quantify the contribution of reformulation to changes in the nutritional quality of consumers’ food puchases | France | 20,000 households, 2008-2011 | Longitudinal | Non-intervention | Nutrition composition | Purchase (expenditure and volume) of food products in four groups – breakfast cereals, biscuits and cakes and potato chips and soft drinks  Market share of products  Key nutrient composition of each product | Kantar | Oqali (French Food Quality Observatory) | Product reformulation programmes have improved the nutritional quality of most food groups examined. Changes were strongest in the saturated fat and sodium content of potato chips and the sodium content of breakfast cereals. These results support the food reformulation as an effective policy compared to promoting behaviour change |
| Stern et al. 2015 | US Household Food Shopping Patterns: Dynamic Shifts Since 2000 And Socioeconomic Predictors. | To identify where US households are shopping for food, to describe how shopping patterns have changed over time and to establish what SES characteristics are associated with shopping patterns | USA | 257,732 households from 2000-2012 | Longitudinal | Non-intervention | Nutrition composition | Household demographics, including ethnicity, age, educational attainment and income  Name and store type of every purchase  Categorisation of households into three groups: primarily grocery cluster, primary mass-merchandiser cluster and combination cluster | Nielsen | N/A | The only trend identified was that low income non-Hispanic black households had a lower probability of belonging to the mass-merchandiser cluster of shopping patterns, otherwise no other association between grocery pattern and ethnicity/income was found. |
| **Author-date** | **Title** | **Objective** | **Geography** | **Demographic** | **Study design** | **Intervention or non-intervention** | **Measuring patterns in food purchases or nutrient content of foods** | **Variables measured and outcomes calculated** | **Source of sales data** | **Source of composition data** | **Summary of key findings** |
| Stern et al. 2016 (1) | The Nutrient Content of U.S. Household Food Purchases by Store Type. | To describe the types of stores US households shop at, to establish if store type changes over time and to establish if the nutrient profile of foods and beverages purchased by US households change by store type | USA | 652,023 household-year observations, 2000-2012 | Longitudinal | Non-intervention | Nutrition composition | Name and store type for every purchase  Energy and nutrient densities (grams of sugar and saturated fat and mg of sodium) per 100g of PFP by store type  Grams of PFP per household per day by store type  Proportion of calories and volume of food and beverage group by store type  Number of calories and volume of food purchased by food type and store type | Nielsen | Gladson Nutrition Database | The number of household purchases of packaged food products increased for mass-merchandisers, convenience stores and warehouse clubs and decreased for grocery chains. The top source of calories from packaged food products was savoury snacks, grain-based desserts and regular soft drinks. |
| Taillie et al. 2016 | Walmart and Other Food Retail Chains: Trends and Disparities in the Nutritional Profile of Packaged Food Purchases. | To better understand the link between food retailer chains, specifically Walmart, and the healthfulness of food purchases | USA | 164,315 households, 2000-2013, fixed effects model | Longitudinal | Non-intervention | Nutrition composition | Mean nutrient densities of household PFPs by retailer, including energy (kcal/100g), total sugar (g/100g), saturated fat (g/100g) and sodium (mg/100g)  Percentage shifts in volumes of food groups purchased  Difference in mean nutrient densities of household PFP by retailer and ethnicity and income | Nielsen | Gladson Nutrition Database (Slining et al. 2013) | The energy, sugar and sodium density of PFPs from Walmart declined from 2000 to 2013, with other stores having more favourable nutritional profile in 2000 but did not shift as much over time. There were also volume declines in purchases of grain-based desserts, savoury snacks and sweets and increases in fruits and vegetables from Walmart. |
| Whybrow et al. 2016 | Buying less and wasting less food. Changes in household food energy purchases, energy intakes and energy density between 2007 and 2012 with and without adjustment for food waste | To test the hypothesis there was no reduction in energy intake once reductions in food waste had been accounted for  Compare the effects of area-based deprivation on these changes | Scotland, UK | 2657 households, 2007-2012 | Longitudinal | Non-intervention | Food purchases | Scottish Index of Multiple Deprivation calculated for each household  Household energy purchases (MJ/adult equivalent per day)  Energy ‘as consumed’ (MJ/adult equivalent per day)  Energy density (kJ/100g) of purchased foods  Energy density (kJ/100g) of foods ‘as consumed’ | Kantar | Kantar | The amount of food energy purchased per adult equivalent decreased from 8.6 MJ/day in 2007 to 8.2 MJ/day in 2012. However, energy intake was not found to be significantly different after accounting for reductions in food waste. |
| Allais et al. 2010 | The effects of a fat tax on French households’ purchases: A nutritional approach | To assess the impact of a fat tax on the nutrients purchased by French households by different income groups | France | 5,000 households | Scenario modelling | Intervention | Food purchases | Price elasticities of 22 food categories by income group  Quantities of 32 nutrients in 22 food categories  Percentage change in total nutrient purchased after 10% fat tax by income group and food group | Kantar | REGAL tables de composition | A fat tax has a small effect on the amount of nutrients purchased by French households and a small impact on bodyweight in the short-term, increasing in the long-term. |
| **Scenario modelling studies** | | | | | | | | | | | |
| Allais et al. 2015 | Mandatory labels, taxes and market forces: An empirical evaluation of fat policies | To identify the causal impact of fat-content labels on consumer choice in the case of yoghurts | France | 13,380 households | Scenario modelling | Intervention | Food purchases | Number of products, mean price and market shares by product type and fat content  Variations in average household annual fat purchases (g) by income and mean BMI and between labelled and taxed products | Kantar | Mintel’s Global New Product Database and magazine *Linéaires.* | In most cases, voluntary FOP fat labels were only found on low-fat products  Results of demand curves suggest that a fat tax has a larger economic impact that mandatory fat FOP-label, but both are equally effective at reducing average fat purchases |
| Dharmasena and Capps 2011 | Intended and unintended consequences of a proposed national tax on sugar-sweetened beverages to combat the US obesity problem | To use a quadratic almost ideal deamand system (QUAIDS) model to estimate the effects of a proposed 20% tax on sugar sweetened beverages (SSBs) | USA | Household size not states; 10 beverage categories used | Scenario modelling | Intervention | Food purchases | Estimated price elasticities by beverage category  Direct, indirect and total effects of 20% tax on each beverage type  Total calories reduced per capita per month and change in body weight | Nielsen | Composition data not used – calories estimated by adapting results of calorie intake data from National Health and Nutrition Examination Survey (NAHNES)^(1)^ | Consumption of isotonic drinks, regular soft drinks and fruit drinks is negatively impacted by the proposed tax, while the consumption of tea, coffee, low-fat milk and fruit juices is positively affected  Reduction in body weight as a result of the tax is estimated to be 1.54 and 2.55 lb per year |
| Finkelstein et al. 2010 | Impact of targeted beverage taxes on higher- and lower-income households | To investigate the impact of a targeted beverage tax on high- and low-income households | USA | Household panel of unspecified size used for a multivariate regression model, 2006 | Scenario modelling | Intervention | Food purchases | Mean daily kcal purchased per capita  Percentage of households that purchased each type of beverages  Average price paid per 1000kcal  Household income quartile  Daily energy consumed per person by beverage categories and by income quartiles  Predicted change in mean energy intake (kcal) by a 20% and 40% tax on both carbonated sugar-sweetened beverages (SSBs) and on all beverages | Nielsen | Gladson Nutriton Database | A 20% and 40% tax on SSBs would reduce calorie intake by 4.2 and 7.8 kcal/d person  Resulting weight loss is estimated at 0.32 kg/person a year and 0.59 kg/person, suggesting that large taxes on SSBs would have a positive influence on weight outcomes in a population. |
| Ford et al. 2015 | Targeted beverage taxes influence food and beverage purchases among households with preschool children | To determine how effective targeted beverage taxes of 10%, 15% and 20% are as a means to reduce purchase of beverages | USA | 14,784 households with one or more children aged 2-5, 2009-2012 | Scenario modelling | Intervention | Food purchases | Socioeconomic characteristics of children aged 2-5 years  Food purchases in grams per capita per day  Elasticities of demand by beverage categories  Change in quantities of beverages purchased based on 10%, 15% and 20% increase in SSBs | Nielsen | Nielsen | Taxes of 10%, 15% and 20% on SSBs were all associated with reduced purchases of juice. Taxes on SSBs plus >1% fat milk and high-sugar milk led to a reduction in calorie purchase from milk, poultry, fish and mixed meats |
| Ford et al. 2017 | SSB taxes and diet quality in US pre-schoolers: estimated changes in the 2010 Healthy Eating Index | To determine how a 20% tax on SSBs might influence the energy intake and diet quality of pre-school children | USA | 2009-2012 data from an unspecified number of households | Scenario modelling | Intervention | Food purchases | Socioeconomic characteristics of children aged 2-5 years  Average prices of products per 100g/ml  Nutrient intakes of children aged 2-5 from the National Health and Nutrition Examination Survey | Nielsen | N/A | 20% increase in price of SSBs was associated with a lower intake of calories (-28kcal/d). Beneficial decreases in empty calories were offset by unfavourable changes in the profiles of saturated fatty acids, protein and fruit and vegetables. This suggests a tax may reduce caloric intake but not improve the overall diet quality |
| Hahn and Davis 2014 | Costs of taxing sodium: A lunch meat application | To measure how much a tax on sodium would affect lunch meat demands and to measure how this impacts consumers’ economic welfare | USA | 25,123 households, 2006 | Scenario modelling | Intervention | Food purchases | Category classification and quantity of each category sold by household  Average price per pound (lb) of product  Mg of sodium per serving  Sodium taxes per pound of lunch meat  Sodium taxes as a % of the average price of lunch meat | Nielsen | USDA nutrition database | A tax rate that increases the price of the highest-sodium lunch meat by 25% can reduce consumption and lower sodium intake from lunch meat by 20% |
| Quirmbach et al. 2018 | Effect of increasing the price of sugar-sweetened beverages on alcoholic beverage purchases: an economic analysis of sales data. | To provide a direct analysis of the relationship between price increases of SSBs and alcoholic drink purchases, by income group | UK | 25,535 households, equating to 6 million beverage purchases | Scenario modelling | Intervention | Food purchases | Mean expenditure on beverages by drink type and household income  Change in demand for beverages based on change of price of sugar-sweetened beverages, by income group | Kantar | Kantar | An increase in the price of high-sugar soft drinks leads to an increase in the purchase of lager  An increase in the price of medium-sugar drinks reduces the purchases of SSBs  An increase in the price of diet soft drinks leads to increased purchases of beer, cider and wine |
| **Author-date** | **Title** | **Objective** | **Geography** | **Demographic** | **Study design** | **Intervention or non-intervention** | **Measuring patterns in food purchases or nutrient content of foods** | **Variables measured and outcomes calculated** | **Source of sales data** | **Source of composition data** | **Summary of key findings** |
| Sharma et al. 2014 | The effects of taxing sugar-sweetened beverages across different income groups | To investigate the impact of SSB taxes on consumption, body weight and tax burden for low-, middle- and high-income groups | Australia | 1390 households | Scenario modelling | Intervention | Food purchases | Quantities of beverages sold by category (L) and household income  Calculated prices (from total expenditure and volume consumed)  Household budget share spent by soft drink category  Price elasticities of beverage type | Nielsen | Nutritional Tables for Australia | A volumetric tax would result in a greater per capita weight loss than a valoric tax (0.41kg vs 0.29kg).  The tax burden of a volumetric tax is also lower for both low- and high-income households compared to the valoric tax.  Therefore the tax burden is lower and the weight reduction is higher under a volumetric tax |
| Tiffin et al. 2014 | The effects of a soft drink tax in the UK. | To investigate the effects of a soft drink tax in the UK, and to differentiate the responsiveness of consumers according to soft drink consumption levels | UK | 24,495 households, 2010 | Scenario modelling | Intervention | Food purchases | Household demographics, including income, age and number of children  Number of portions of soft drinks purchased per week  Shares and total expenditure on food and drink  Unconditional own-price elasticities for drinks  Percentage change in quantity consumed | Kantar | N/A | A tax on soft drinks would have the largest impact on households with high levels of soft drink consumption and that substitution may mitigate effects. The overall impact of a soft drink tax on consumption is likely to be small. |
